# miRNAs Contained in Extracellular Vesicles Cargo Contribute to the Progression of Idiopathic Pulmonary Fibrosis: An In Vitro Aproach

**DOI:** 10.3390/cells11071112

**Published:** 2022-03-25

**Authors:** Jovito Cesar Santos-Álvarez, Juan Manuel Velázquez-Enríquez, Rosendo García-Carrillo, César Rodríguez-Beas, Alma Aurora Ramírez-Hernández, Edilburga Reyes-Jiménez, Karina González-García, Armando López-Martínez, Laura Pérez-Campos Mayoral, Sergio Roberto Aguilar-Ruiz, María de los Ángeles Romero-Tlalolini, Honorio Torres-Aguilar, Luis Castro-Sánchez, Jaime Arellanes-Robledo, Verónica Rocío Vásquez-Garzón, Rafael Baltiérrez-Hoyos

**Affiliations:** 1 Facultad de Medicina y Cirugía, Universidad Autónoma Benito Juárez de Oaxaca, Oaxaca 68120, Mexico; jovitocesarsa@hotmail.com (J.C.S.-Á.); juanmanuelvela_enriquez@live.com (J.M.V.-E.); aramih_09@hotmail.com (A.A.R.-H.); edilreyesjimnez@yahoo.com.mx (E.R.-J.); k.igg@hotmail.com (K.G.-G.); armandoloopez37@gmail.com (A.L.-M.); sar_cinvestav@hotmail.com (S.R.A.-R.); 2 Centro Universitario de Investigaciones Biomédicas, Universidad de Colima, Colima 28045, Mexico; rosendo_garcia@ucol.mx; 3 Departamento de Física, Universidad de Sonora, Hermosillo 83000, Mexico; cesar.rodriguez@unison.mx; 4 Centro de Investigación, Facultad de Medicina, Universidad Autónoma “Benito Juárez” de Oaxaca, Oaxaca 68120, Mexico; laupcm9@gmail.com; 5 CONACYT-Facultad de Medicina y Cirugía, Universidad Autónoma Benito Juárez de Oaxaca, Oaxaca 68120, Mexico; romerotlalolini@gmail.com (M.d.l.Á.R.-T.); veronicavasgar@gmail.com (V.R.V.-G.); 6 Facultad de Ciencias Químicas, Universidad Autónoma Benito Juárez de Oaxaca, Oaxaca 68120, Mexico; qbhonorio@hotmail.com; 7 Centro Universitario de Investigaciones Biomédicas, CONACYT-Universidad de Colima, Universidad de Colima, Colima 28045, Mexico; luis_castro@ucol.mx; 8 CONACYT-Instituto Nacional de Medicina Genómica, Ciudad de México 14610, Mexico; jarellanes@inmegen.gob.mx

**Keywords:** idiopathic pulmonary fibrosis, extracellular vesicles, fibroblasts, small-RNA seq

## Abstract

Idiopathic pulmonary fibrosis (IPF) is a chronic, progressive lung disease. Lesions in the lung epithelium cause alterations in the microenvironment that promote fibroblast accumulation. Extracellular vesicles (EVs) transport proteins, lipids, and nucleic acids, such as microRNAs (miRNAs). The aim of this study was to characterize the differentially expressed miRNAs in the cargo of EVs obtained from the LL97 and LL29 fibroblast cell lines isolated from IPF lungs versus those derived from the CCD19 fibroblast cell line isolated from a healthy donors. We characterized EVs by ultracentrifugation, Western blotting, and dynamic light scattering. We identified miRNAs by small RNA-seq, a total of 1144 miRNAs, of which 1027 were known miRNAs; interestingly, 117 miRNAs were novel. Differential expression analysis showed that 77 miRNAs were upregulated and 68 were downregulated. In addition, pathway enrichment analyses from the Gene Ontology and Kyoto Encyclopedia of Genomes identified several miRNA target genes in the categories, cell proliferation, regulation of apoptosis, pathways in cancer, and proteoglycans in cancer. Our data reveal that miRNAs contained in EVs cargo could be helpful as biomarkers for fibrogenesis, diagnosis, and therapeutic intervention of IPF.

## 1. Introduction

Idiopathic pulmonary fibrosis (IPF) is a chronic interstitial disease characterized by a radiographic or histologic pattern known as unusual interstitial pneumonia [1]. The median survival time for IPF patients is 3 to 5 years [2]. Environmental and genetic factors are significant contributors to IPF pathogenesis, and it is well known that persistent micro-injuries in the alveolar epithelium may be the predisposing insult that disrupts the cellular repair process [3]. IPF is a fibrotic disease characterized by the accumulation of fibroblasts proliferation and activation, resulting in collagen synthesis and deposition of extracellular matrix (ECM) proteins and glycoproteins [4]. In a normal wound repair process, fibroblasts produce ECM but are effectively controlled through apoptosis activation [5]. However, during IPF progression, a significant accumulation of fibroblast is promoted by the cell microenvironment [6]. 

Cellular communication is characterized by cell–cell junctions, adhesion contacts, and soluble factors [7]. Mainly, extracellular vesicles (EVs) perform central roles in the pathogenesis of IPF, as they transport and transfer diverse proteins, lipids, mRNAs, and microRNA (miRNA) [8,9]. miRNAs are small non-coding RNAs, generally comprising 19–22 nucleotides, and they are complementary to target sequences of mRNAs [10]. It has been estimated that they can modulate the expression of more than 50% of protein-coding genes involved in cellular processes, such as apoptosis, proliferation, and differentiation [11].

Previous reports have suggested that miRNAs participate in IPF fibrogenesis since they have been found to be dysregulated in the tissue of patients [12,13,14]. For instance, upregulation of miR-21 and miR-101 is associated with IPF progression [15,16]. Besides, a significant increase of miR-21 and miR-155 expression has been found in serum from IPF patients [17]. However, a miRNAs profile within EVs secreted during IPF fibrogenesis has not yet been identified. On the other hand, recent studies have demonstrated that RNA sequencing (RNA-seq) is a valuable technology to identify specific features, such as differential splicing in the lung tissue [18], revealing that the lung epithelial is composed of subpopulations of cells [19]; additionally, this technology has also shown that different signaling pathways may be contributing to the IPF development [20]. 

The aim of this study was to characterize the differentially expressed miRNAs in the cargo of EVs obtained from the LL97 and LL29 fibroblast cell lines isolated from IPF lungs versus those derived from the CCD19 fibroblast cell line isolated from a healthy donor. Using RNA-seq technology, we identified differentially expressed miRNAs (DE-miRNA) contained within EVs cargo derived by fibroblast cell lines isolated from healthy and IPF patients. Our findings provide evidence that miRNAs contained in EVs might help reveal novel diagnostic biomarkers and molecular mechanisms of IPF progression.

## 2. Materials and Methods

### 2.1. Cell Culture

The human lung fibroblast cell lines LL29 and LL97A, isolated from IPF patients, were obtained from the American Type Culture Collection (ATCC, Cat. No. CCL-134 and CCL-191, respectively; Manassas, VA, USA), and CCD19 cells were considered as normal control (ATCC, Cat. No. CCL-210; Manassas, VA, USA). All cells were cultured in Eagle’s minimal essential medium (EMEM; ATCC No. 30-2003; Manassas, VA, USA) supplemented with 10% FBS (26140079, Gibco, Thermo Fisher Scientific, Waltham, MA, USA) and 100 U/mL penicillin/streptomycin (15140122, Gibco, Thermo Fisher Scientific, Waltham, MA, USA). Experiments were performed using passages from 8 to 12, and cells were grown in 10 cm culture plates in a humidified incubator at 37 °C and 5% CO_2_.

### 2.2. Isolation of Extracellular Vesicles

EVs were obtained from the culture supernatant of LL97 (IPF-1), LL29 (IPF-2), and CCD19 (NL) cell lines using ultracentrifugation. Briefly, cells lines were grown in 10 cm diameter plates to 70% confluence. Then, cells were washed three times with PBS before adding fresh medium supplemented with 5% FBS EVs-reduced, which was used to avoid RNA contamination contained in EVs from FBS. After incubation for 48 h, culture media were collected and differentially centrifuged to remove cell debris and apoptotic bodies, first at 2000× *g* for 30 min and then at 12,000× *g* for 30 min, in both centrifugation rounds at 4 °C. Subsequently, EVs were extracted by centrifugation at 100,000× *g* for 3 h at 4 °C in a TH641 rotor (Thermo Scientific, Waltham, MA, USA). Next, EVs were resuspended in 150 μL of sterile PBS (70011044, Gibco, Thermo Fisher Scientific, Waltham, MA, USA) and filtered for particle size determination. Protein extraction was performed by resuspending the EVs in 150 μL of CHAPS lysis buffer supplemented with proteases inhibitors. For RNA isolation, 700 μL of Qiazol (79306, Qiagen, Germantown, MD, USA) were added.

### 2.3. Dynamic Light Scattering (DLS) Analysis

Once EVs were isolated, the size distribution was determined by dynamic light scattering measurements (DLS) in a Malvern Zetasizer NanoZS equipment (Malvern Instruments, Malvern, UK), with a resolution set at 0.5 ηm and sensitivity at 0.1 ppm, at 25 °C. 

### 2.4. Western Blot

Proteins extracted from EVs were subjected to Western blot analysis to identify EV markers. Briefly, SDS-PAGE electrophoresis was performed in 10% polyacrylamide gels and then transferred onto the PVDF membrane. Then, membranes were blocked and incubated with primary antibodies Anti-HSP90 αβ and Anti Alix (1:500, SC-13119 and SC-53540, 1:500, respectively; Santa Cruz Biotechnology, Inc. Dallas, TX, USA), and Anti Flotilin-1 (1:500, BD-610821, Franklin lakes, NJ, USA). Then, membranes were incubated with horseradish peroxidase-conjugated anti-mouse secondary antibodies (1:5000; SC-516102; Santa Cruz Biotechnology, Inc. Dallas, TX, USA). Finally, protein bands were visualized with a 1-Step Ultra TMB-Blotting reagent (37574, Thermo Scientific, Waltham, MA, USA).

### 2.5. Isolation of Small RNA from EVs

Pellets of EVs isolated from cell lines were resuspended in 700 μL of Qiazol (79306, Qiagen, Germantown, MD, USA.) and incubated for 5 min at room temperature (RT). Next, 140 μL of chloroform were added (C7559, Sigma, Burlington, MA, USA), incubated 5 min at RT, then centrifuged at 12,000× *g* for 15 min at 4 °C. The aqueous phase was separated into a clean microtube, and then 525 μL of 100% ethanol were added (E7023, Sigma, Burlington, MA, USA) and centrifuged at 8000× *g* for 1 min at RT. Then, 700 μL of RWT buffer were added, centrifuged at 8000× *g* for 1 min at RT, the excess was decanted, 500 μL of RPE buffer were added, and centrifuged at 8000× *g* for 1 min at RT. Subsequently, supernatants were discarded, and 500 μL of RPE buffer were added and centrifuged at 8000× *g* for 2 min at RT. Then, the column was transferred to a collection tube, and 20 μL of RNase-free water were added and centrifuged at 8000× *g* for 1 min at 4 °C in order to elute the RNA. Finally, RNA was quantified by using the Quantus™ Fluorometer equipment (Promega, Madison, WI, USA).

### 2.6. Small RNA Sequencing

MiRNAs from EVs were evaluated in duplicate and processed using the following methodology. First, RNA degradation and contamination were assessed on a 1% agarose gel and quantified on the NanoPhotomer^®^ spectrophotometer (IMPLEN, Westlake, CA, USA). Then, RNA integrity was assessed by using the RNA Nano 6000 Assay Kit and the Agilent Bioanalyzer 2100 system (Agilent Technologies, Santa Clara, CA, USA).

### 2.7. Library Preparation for miRNAs Sequencing

One μg of total RNA from EVs samples was used to generate an RNA library using the NEBNext Multiplex Small RNA Library Prep Set for Illumina (Illumina, San Diego, CA, USA). PCR amplification was performed using LongAmp Taq 2X Master Mix, SR Primer (Illumina, San Diego, CA, USA), and index primer. PCR products were purified using an 8% polyacrylamide gel (100 V, 80 min). DNA fragments of ~140–160 bp (the length of non-coding small RNA plus the 3′ and 5′ adaptors) were recovered and dissolved in 8 μL of elution buffer. Finally, library quality was assessed using the Agilent Bioanalyzer 2100 system and susceptible DNA chips.

### 2.8. Clustering and Sequencing

Clustering of the index-coded samples was performed using a cBot cluster generation system, as well as the TruSeq SR v3-cBot-HS cluster kit (Illumina). After cluster generation, the library was sequenced on an Illumina platform (NovaSeq 6000), and then 50 bp single-end reads were generated.

### 2.9. Quality Control

Raw reads from fastq format were first processed using custom perl and python scripts. Briefly, clean reads were obtained, and contaminants were discarded; in addition, Q20, Q30, and GC contents were calculated, and then, the length range of clean reads was selected for further analysis.

### 2.10. Alignment of Known miRNAs and Novel miRNAs Prediction

For mapping known miRNAs, small tags were generated, and software such as mirdeep2 [21] and miRBase 20.0 was used (accessed 2 June 2021). We used miREvo [22] and mirdeep2 [21] software to generate both a secondary structure and the minimum free energy of small RNA tags not annotated in previous steps.

### 2.11. Quantification and DE-miRNAs

For normalizing the reading counts and appropriate depth, we used transcripts per million (TPM) using the following conditions: Normalization formula: Normalized expression = mapped read counts/total reads × 1,000,000. To perform differential expression analysis, including two replicates, we used the DESeq R package version (1.8.3) [23]. *p*-value was adjusted using the Benjamini and Hochberg method. The corrected *p*-value of ˂0.05 was set as the cutoff for significantly predetermined differential expression.

### 2.12. miRNAs Target Gene Analysis

To determine miRNAs target genes showing the highest change value, we used miRWalk (http://mirwalk.umm.uni-heidelberg.de/, accessed on 2 September 2021) as well as the miRDB database (http://mirdb.org/miRDB/, accessed on 2 September 2021), an online database for miRNAs target prediction genes. Although each miRNA could target hundreds of genes, the analysis was limited to the three most abundant exosomal miRNAs, both upregulated and downregulated, and shared between both samples.

### 2.13. Real-Time RT-PCR

miRNAs were extracted with the miRNeasy kit (Qiagen, Hilden, Germany) following the manufacturer’s instructions and reverse transcription was performed with the miScript II RT kit (Qiagen, Hilden, Germany). Subsequently, complementary DNAs (cDNAs) were amplified from the following 6 miRNAs (hsa-miR-4326, hsa-miR-200c-3p, hsa-miR-122-5p, hsa-miR-615-3p, hsa-miR-181a-2-3p, and hsa-miR-143-3p) using the miScript miRNA qPCR (Qiagen, Hilden, Germany).The following thermal cycling conditions were used: 95 °C for 15 min, followed by 40 cycles of 94, 55, and 70 °C for 15, 30, and 30 s, respectively. Data were analyzed using PCR array data analysis tools (Bio-Rad). Each sample was detected three times.

### 2.14. Bioinformatics Annotations

The InteractiVenn web application (http://www.interactivenn.net/; accessed on 1 September 2021) was used to perform a 3-way Venn diagram [24]. miRNA target gene candidates were subjected to gene ontology (GO) enrichment analysis and included three categories, namely biological process (BP), molecular function (MF), cellular component (CC), and it was performed online using the gene annotation co-occurrence discovery classification system (GeneCodis; https://genecodis.genyo.es/; accessed on 5 September 2021) [25]. The database resource Kyoto Encyclopedia of Gene and Genome (KEGG) from human genome sequencing was used to study the functions of a biological system and was performed online using the gene annotation co-occurrence discovery classification system (GeneCodis; (https://genecodis.genyo.es/; accessed on 5 September 2021) [25]. GO, and KEGG analyses were considered significant when *p* < 0.05. Cytoscape v3.8.2 software (Cytoscape Consortium, San Diego, CA, USA) was used to visualize both upregulated and down-regulated miRNA interaction networks shared between samples [26].

## 3. Results

### 3.1. Validation of EVs Derived from Fibroblasts

**Figure 1:**
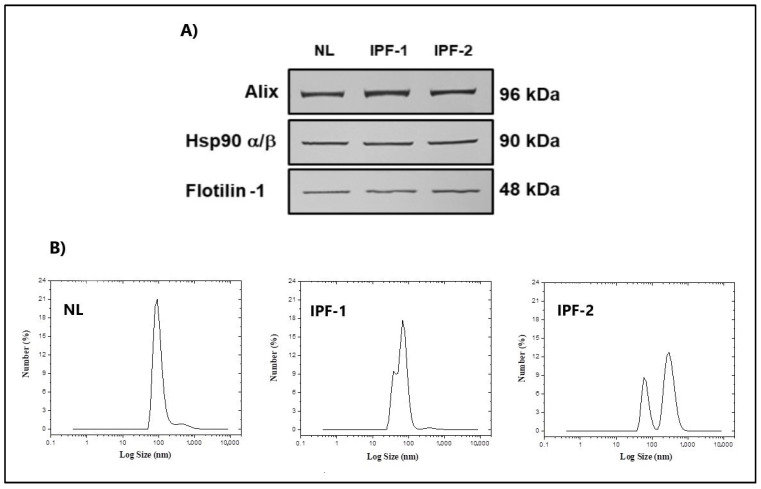
Validation of EVs from healthy and IPF lung fibroblasts. (**A**) Western blot EVs protein markers including ALIX, flotillin, and Hsp90. (**B**) Dynamic light scattering analysis of EVs for size terminations. Assays were performed in triplicate.

We used standard isolation protocol through sequential ultracentrifugation to validate EVs isolated from NL, IPF-1, and IPF-2 cell cultures [27]. EVs were validated by detecting surface markers and size distribution. Western blotting analysis confirmed that proteins, such as Alix, Flotilin-1, and Hsp90, were enriched in isolated EVs samples (Figure 1A). We next performed dynamic light scattering analysis, showing that the average particle size value was 106.4 nm for NL and 67.61 nm for IPF-1, but for IPF-2 have observed two values, one at 66.99 and another at 414.7 nm, this finding is very interesting, because it suggests 2 subpopulations of extracellular vesicles, which would need more assays to confirm. These results confirmed that sequential ultracentrifugation is a valid and efficient method to isolate EVs between 30 and 1000 nm diameter (Figure 1B and Table S1).

### 3.2. High-Throughput Sequencing of miRNAs

Deep sequencing to construct small RNA libraries was performed using the Illumina platform. We identified 22,669,187 raw reads from 2 replicates per EVs sample, with an average of 53.09% of GC content, as well as an average of 97.87% clean reads in the appropriate 19–22 nucleotide sizes with no low-quality reads (Table S2).

### 3.3. miRNAs Expression Profiling of EVs Isolated from Lung Fibroblasts

**Figure 2:**
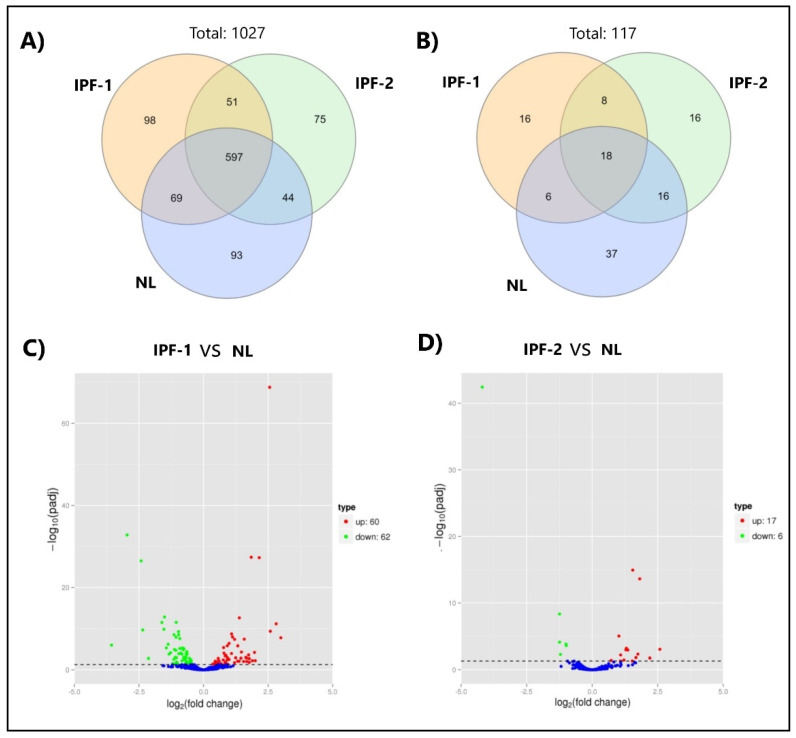
miRNAs expressed in EVs of IPF fibroblasts. (**A**) Venn diagram shows known miRNAs shared in IPF-1, IPF-2, and NL groups. (**B**) Venn diagram shows novel miRNAs shared in IPF-1, IPF-2, and NL groups. (**C**,**D**) Volcano plots of differentially expressed miRNAs within EVs from IPF-1 vs. NL and IPF-2 vs. NL. The *x*-axis shows the fold change of miRNAs expression, and the *y*-axis shows the statistical significance (*p* adjusted threshold < 0.05), The dot representing each miRNA, blue dots indicate no significant difference miRNA, red dots indicate significant upregulation of miRNA, green dots indicate significant difference by miRNA.

Using next-generation RNA-seq, we identified miRNAs contained in EVs from IPF fibroblast. The analysis detected 1027 miRNAs already identified by homology with known sequences available in mirBase, as well as 117 novel miRNAs (Table S3). Venn diagram shows that 597 miRNAs are shared between IPF-1, IPF-2, and NL groups, 51 miRNAs are exclusively shared between IPF-1 and IPF-2, and 93 miRNAs are expressed in NL only (Figure 2A). RNA-seq analysis predicts new miRNAs, using the hairpin structure of a precursor a new miRNA can be predicted, using miREvo [22] and mirdeep2 [21] software, 117 new miRNAs including their secondary structures were predicted. The Venn diagram shows that 18 novel miRNAs are shared across all groups of EVs, 37 novel miRNAs are expressed only in NL, and 8 novel miRNAs are common among IPF-1 and IPF-2 groups (Figure 2B and Table S4). 

### 3.4. Differentially Expressed miRNA within EVs from Lung Fibroblasts

Differential expression profiling was performed by comparing the data obtained from small RNA sequencing of IPF-1, IPF-2, and NL groups. Based on q-value < 0.01 and |log2foldchange| > 1 criterion, we found that the comparison between IPF-1 and NL group showed 60 and 62 miRNAs upregulated and downregulated, respectively (Figure 2C, Tables S5 and S6), while the comparison between IPF-2 and NL group showed 17 and 6 miRNAs upregulated and downregulated, respectively (Figure 2D, Tables S5 and S7). Table 1 and Table S8 show that 12 miRNAs reached the highest upregulated value and 5 of them the lowest downregulated ones. Some of the most elevated upregulated miRNAs were miR-4326, miR-200c-3p, and miR-122-5p, and some of the lowest downregulated miRNAs were miR-615-3p, miR-181a-2-3p, and miR-143-3p. In addition, 2 novel miRNAs, namely 104 and 85, were statistically significant too. This result shows that these miRNAs may be playing a relevant role in the EVs-dependent gene expression regulation.

### 3.5. Gene Ontology Enrichment Analysis of EVs miRNAs from IPF Fibroblasts

To determine the effects of DE-miRNAs in IPF, we then identified the associated functional roles of those miRNAs based on gene ontology (GO) terms by including the following categories: Biological Process (BP), Cellular component (CC), and Molecular Functions (MF). Thus, to get additional information on miRNAs expression profiles, we performed an online GO enrichment analysis using the gene annotation co-occurrence discovery classification system (GeneCodis; https://genecodis.genyo.es/ accessed on 5 September 2021) [25].

**Figure 3:**
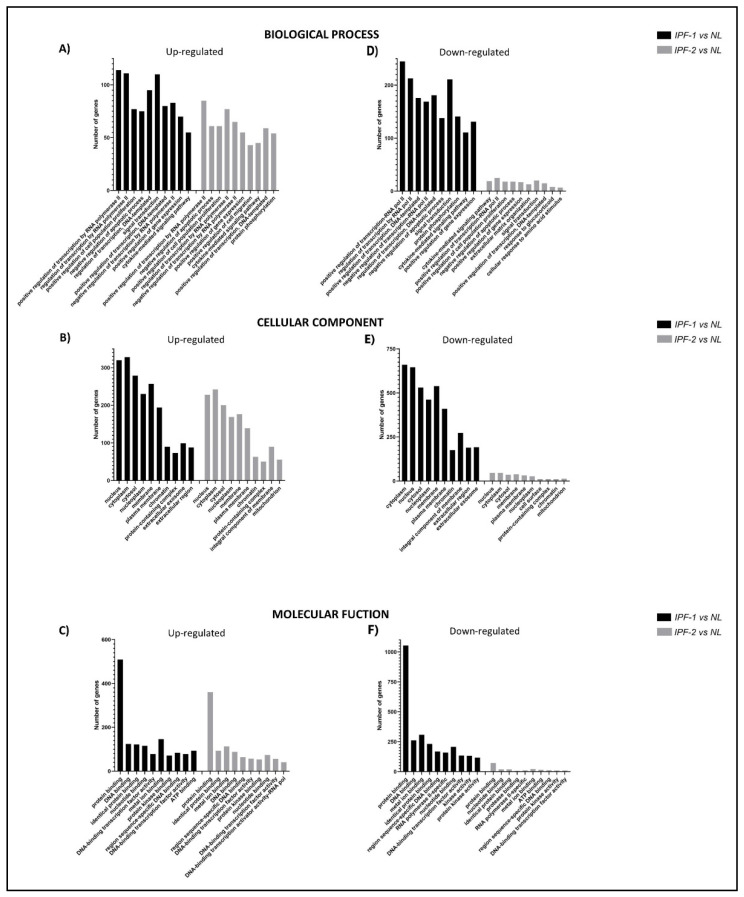
Gene ontology enrichment analysis of target genes regulated by DE-miRNAs in EVs. Using the top DE-miRNAs, gene ontology was determined using the GeneCodis platform. (**A**–**C**) Top 10 target gene terms in Biological Processes, Cellular Components, and Molecular Functions category for upregulated miRNAs. (**D**–**F**) 10 classes with the highest number of target genes in GO categories for downregulated miRNAs. The *x*-axis shows GO terms of ontologies, and the *y*-axis indicates the number of target genes annotated in GO terms.

Genes found as possible targets of upregulated miRNAs in the BP category were: methyl-CpG binding protein 2 (MECP2), disheveled segment polarity protein 2 (DVL2), G protein subunit alpha i2 (GNAI2), C-X-C motif chemokine ligand 12 (CXCL12), and AT-rich interaction domain 1A (ARID1A), which correspond to categories such as positive regulation of transcription by RNA polymerase II, cell proliferation, and signal transduction (Figure 3A and Table S9). In CC, the genes found were: myosin light chain kinase (MYLK), DNA methyltransferase 3 alpha (DNMT3A), sirtuin 1 (SIRT10), which correspond to categories such as cytoplasm, nucleus, and chromatin (Figure 3B and Table S9). In MF, the genes found were: DIRAS family GTPase 3 (DIRAS3), DNA methyltransferase 3 alpha (DNMT3A), and ATP binding cassette subfamily G member 1 (ABCG1), which correspond to categories such as protein binding, DNA, and ATP (Figure 3C and Table S9).

The genes found as possible targets of downregulated miRNAs in the BP category were: caspase 3 (CASP3), mitogen-activated protein kinase 7 (MAPK7), prostaglandin-endoperoxide synthase 2 (PTGS2); these belong to categories such as cytokine signaling, regulation of transcription by RNA pol II and regulation of apoptosis (Figure 3D and Table S10). In CC, the genes found were: hexokinase 2 (HK2), prostaglandin-endoperoxide synthase 2 (PTGS2), integrin subunit beta 3 (ITGB3); these belong to categories such as a nucleus, cytoplasm, and protein complex (Figure 3E and Table S10). In MF, the genes found were: tumor necrosis factor (TNF), collagen type III alpha 1 chain (COL3A1), and hexokinase 2 (HK2); these belong to categories such as binding to proteins, nucleotides, and proteases (Figure 3F and Table S10). 

### 3.6. KEGG Enrichment Analysis of miRNAs from EVs of IPF Fibroblasts

**Figure 4:**
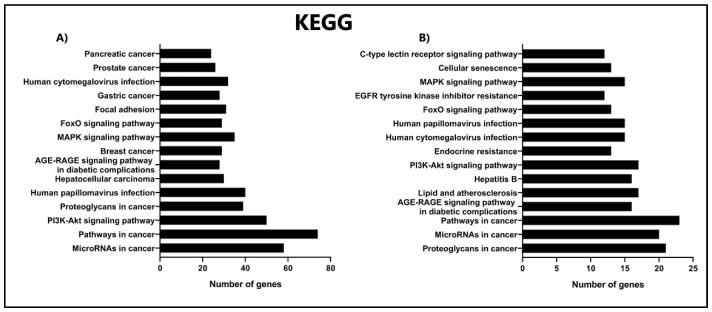
Kyoto Encyclopedia of Genes and Genomes (KEGG) enrichment analysis. KEGG pathway of DE-miRNAs (**A**) upregulated and (**B**) downregulated. The *x*-axis indicates the number of target genes, and the *y*-axis shows pathway names.

KEGG is a database for performing a systematic analysis of gene functions [28]. Here, we used the hypergeometric test and FDR correction method of Benjamini and Hochberg for performing KEGG analysis. Our results indicate that miRNAs target genes for upregulated and downregulated expression belong to the following classes: Pathways in cancer (STAT5A, MMP1, and CXCL12), microRNAs in cancer (HMOX1, GNAI2, and DVL2), proteoglycans in cancer (NRAS, DNMT3A, and CASP3), PI3K-Akt signaling pathway (ITGB4, ITGB3, and MTOR), and FoxO signaling pathway (STAT3, TGFBR1, and IL6). These results suggest that these genes expression could be affected by miRNAs transported in EVs cargo during the IPF development (Figure 4A,B, Tables S11 and S12).

### 3.7. DE-miRNA Target Gene Analysis

**Figure 5:**
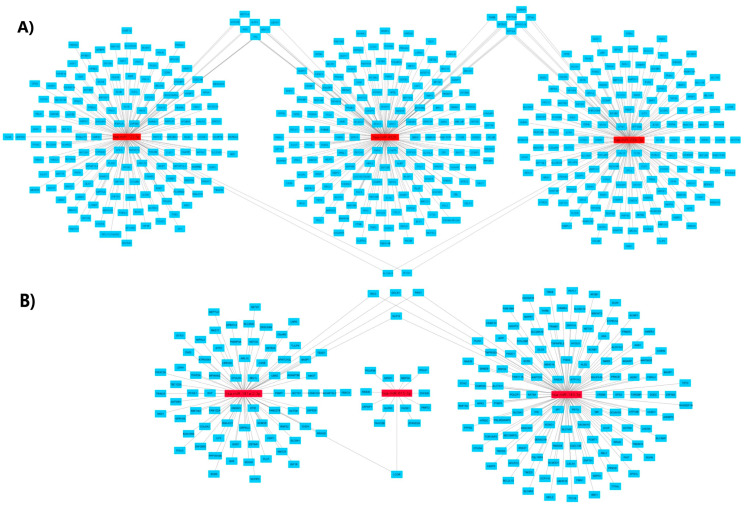
Interaction networks of upregulated and downregulated miRNA target genes. (**A**) upregulated miRNAs, (**B**) downregulated miRNAs. Cytoscape program was used to create the interaction network, using miRDB as a database and miRWalk website for analysis.

The MiRWalk 2.0 database was used to predict targets genes for three upregulated and three downregulated miRNAs (Table 1). Our analysis showed that upregulated miRNAs, namely miR-4326, miR-200c-3p, miR-122-5p, a total of 171, 158, and 150 genes, respectively, were as found their targets. On the other hand, downregulated miRNAs, namely, miR-615-3p, miR-181a-2-3p, and miR-143-3p, a total of 13, 79, and 126 genes, respectively, were found as their targets (Figure 5B and Tables S13 and S14). 

### 3.8. KEGG Enrichment Analysis of Target Genes from Hub miRNAs

**Figure 6:**
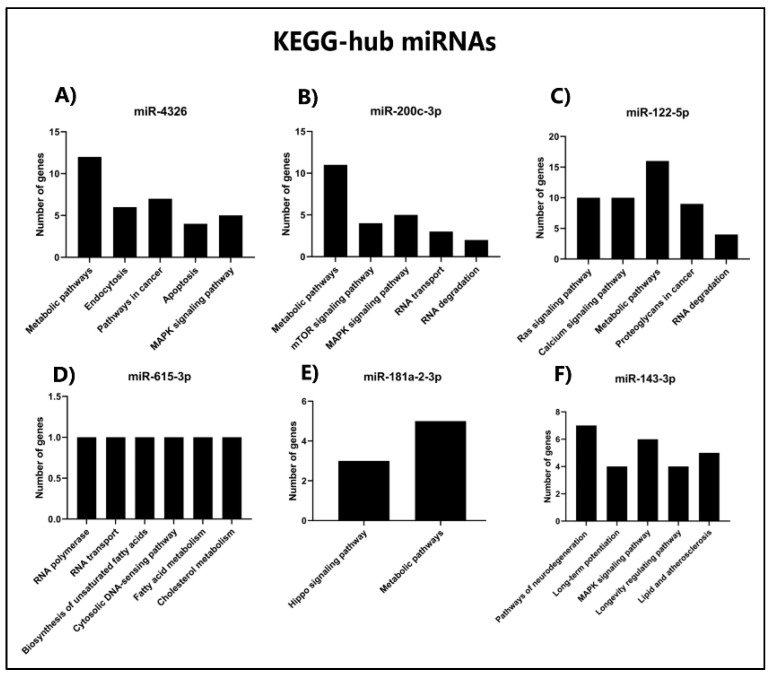
KEGG enrichment analysis of target genes from hub miRNAs. Upregulated miRNAs, (**A**) miR-4326, (**B**) miR-200c-3p, (**C**) miR-122-5p; downregulated miRNAs, (**D**) miR-615-3p (**E**), miR-181a-2-3p, and (**F**) miR-143-3p. The *x*-axis shows pathway names, and the *y*-axis indicates the number of target genes.

We performed KEGG enrichment analysis of the target genes of hub miRNAs to determine the cellular pathways involved in IPF. Our results indicated that the target genes of hub miRNAs for upregulated expression enriched metabolic pathways, mTOR signaling pathway, and proteoglycans in cancer. Some genes related to these pathways are malonyl-CoA-acyl carrier protein transacylase (MCAT), eukaryotic translation initiation factor 4E (EIF4E), and protein kinase cAMP-activated catalytic subunit beta (PRKACB) (Figure 6A–C and Table S15). For the downregulated core miRNA target genes, RNA transport, Hippo signaling pathway, and MAPK pathway were enriched. Some genes related to these pathways are nucleoporin 62 (NUP62), angiomotin (AMOT), and mitogen-activated protein kinase 7 (MAPK7). Interestingly, for miR-181a-2-3p, 79 genes were found, classified in only 2 categories using the Genecodis resource, which may suggest specific pathways for its action (Figure 6D–F and Table S15). In addition, these results suggest the probable processes that the hub miRNAs can regulate in the IPF pathogenesis.

### 3.9. Validation of the Expression of Selected DE-miRNAs by qRT-PCR

**Figure 7:**
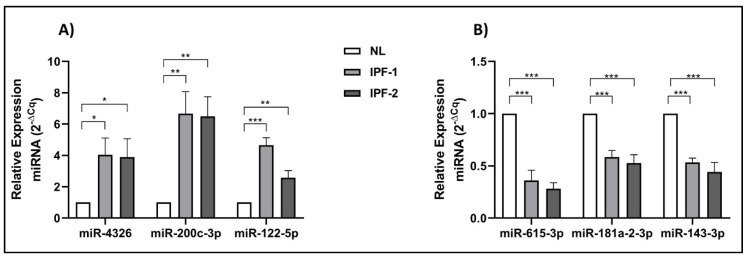
Hub DE-miRNAs were validated by performing quantitative RT-PCR. The relative expression levels of (**A**) 3 up regulated miRNAs (miR-4326, miR-200c-3p, and miR-122-5p) and (**B**) 3 down regulated miRNAs (miR-615-3p, miR-181a-2-3p, and miR-143-3p) in EVs from fibroblast with IPF and healthy, were determinated by qRT-PCR. Compared with the expression basal from healthy miRNAs, 6 miRNAs were significantly higher and lower, respectly. (*, **, *** *p* < 0.05).

To validate the expression of the hub DE-miRNAs, qRT-PCR was used. We found that the expression of miR-4326, miR-200c-3p, and miR-122-5p was significantly increased in fibroblast vesicles with IPF1 and IPF2 (*p* < 0.05) (Figure 7A). In addition, the expression of miR-615-3p, miR-181a-2-3p, and miR-143-3p was found to be significantly reduced in fibroblast vesicles with IPF1 and IPF2 (*p* < 0.05) (Figure 7B). These data are in agreement with those found by small RNA-seq. Furthermore, suggest the presence of a differential expression pattern in the EVs of fibroblasts.

## 4. Discussion

IPF, the most common form of idiopathic interstitial pneumonia (IIP), is characterized by increasing fibroblast proliferation and activation, including collagen synthesis and deposition of ECM proteins and glycoproteins [29]. These processes are conducted by over-activating intercellular communication in the lung microenvironment through cytokines production, cell junctions, and EVs participation, which play an essential role in the disease progression [30]. EVs can transport miRNAs, a class of non-coding small RNAs that are approximately 20 nucleotides in length and are critical players in gene expression regulation [31]. It has been reported that miRNAs are involved in IPF pathogenesis and could be helpful for the diagnosis or prognosis of the disease [32]. The present study aimed to characterize the differentially expressed miRNAs contained within EVs cargo.

In the present study, we identified DE-miRNAs in EVs cargo isolated from human IPF fibroblast cell lines that may play a key role in the progression of IPF. The results shown in this study may provide new insights into the molecular mechanisms of IPF. However, some limitations of this investigation, such as the need for additional experimental evidence that can validate the involvement of the identified miRNAs either in vivo systems or in samples from patients bearing IPF, have yet to be addressed. Also, the association of miRNAs in EVs in other cells, such as macrophages, alveolar epithelial cells, and stem cells, should be investigated to confirm the findings. 

The use of cell lines may represent the pathogenesis state of the individual from which it was drawn; one study reported to investigate the CC 7 chemokine receptor (CCR7) in PII biopsies—they cultured primary fibroblasts grown from idiopathic pulmonary fibrosis in CB-17 mice and were able to observe that their cultures could induce interstitial fibrosis and increased hydroxyproline [33]. 

The study of miRNA in patients with IPF is relevant because it may reflect biological states that may be associated with its progression; some studies have focused on looking at this expression at the serum level, it being observed that there is a differential expression, miR-21 and miR-101-3p could correlate with clinical-pathological characteristics of patients [34]. Evaluating miRNA in EVs can give us a similar overview and propose a role for these miRNAs in IPF.

This study performed small RNA sequencing to identify miRNAs contained in EVs from IPF and healthy fibroblasts. We found that EVs had 1027 known miRNAs, already reported in the miRBase database, but additionally, our next-generation sequencing analysis identified 117 novel miRNAs in the EVs cargo. In addition, we determined the DE-miRNA by comparing IPF-1 and IPF-2 samples with the NL group, and we found 60 and 17 upregulated miRNAs and 68 and 6 downregulated miRNAs, respectively. This result indicated that miRNAs in the EVs cargo might be involved in pulmonary fibrosis development. The hub miRNA with the highest differential expression was miR-4326, which was upregulated in EVs secreted by IPF fibroblasts compared with EVs derived of normal fibroblasts. Furthermore, miR-615-3p was the most downregulated miRNA in EVs of IPF fibroblasts. Gene Ontology analysis showed that the most representative terms in the category BP, CC, and MF, these target genes that the hub miRNAs can influence, and in the future might offer a response to the interaction with these miRNAs, and thus modify cellular processes that may contribute to disease fibrogenesis.

The hub miRNAs miR-4326, miR-200c-3p, and miR-122-5p, which showed the highest expression levels, have not yet been studied in IPF. This may be since we perform small RNA deep sequencing of EVs cargo from cell lines bearing IPF. However, miR-4326 is upregulated in lung tumor tissue; moreover, its upregulation promotes lung cancer cell proliferation. Also, miR-4326 targeted the 3’UTR of tumor suppressor adenomatous polyposis coli 2 (APC2), a negative regulator of the Wnt pathway. Thus, the low expression of APC2 and the increase of miR-4326 in lung cancer cell proliferation confirm that miR-4326 stimulates cell proliferation by inhibiting APC2 [35]. Additionally, our KEGG enrichment analysis of miR-4326 target genes showed pathways such as apoptosis and MAPK signaling pathways (Figure 6A); these pathways have been linked to the stimulus involved in the development of pulmonary fibrosis [36]. Besides, the metabolic pathways, a KEGG pathway regulated by miR-4326, have been studied in lung tissue of patients with IPF, where they have found altered energy and fatty acid metabolism, which could guide us as to whether these miRNAs may contribute to the modification of this metabolism, which may contribute to the pathogenesis of IPF [37].

The miR-200c-3p has been studied in exosomes derived from the oral squamous cell carcinoma lines SQUU-A and SQUU-B, and silencing this miRNA reduced the invasive capability of these cells [38]. Another report has proposed that upregulation of miR-200c-3p might be helpful as a biomarker for prostate cancer [39]. However, the miR-200c family has been found upregulated and downregulated in metastasis processes from primary tumors such as colon, bladder, breast, and lung cancer [40]. In addition, miR-200c-3p and miR-203a-3p have been proposed as potential regulators of the resistance to epidermal growth factor receptor (EGFR) and tyrosine kinase inhibitors (TKIs) by modulating the epithelial-to-mesenchymal transition process through apoptosis in non-small-cell lung cancer (NSCLC) [41]. Our KEGG pathway analysis of miR-200c-3p target genes showed MAPK and mTOR signaling pathways (Figure 6B), which have been studied, the p38 MAPK pathway has been studied in macrophages, Forkhead Box M1 (FOXM1) expression has been observed in IPF patients, in a mouse model deficient in FOXM1, an exacerbation of fibrosis was found, it was found that the deletion of this transcription factor activated the MAPK pathway, increasing fibrosis [42]. 

The miR-122-5p has been found upregulated in the serum of patients with fatty liver disease, associated with lipid metabolism [43]. Bioinformatic analysis has detected it as a pancreatic cancer marker candidate, and qPCR analysis has confirmed significant upregulation of miR-122-5p, miR-1273g-3p, and miR-6126 [44]. MiR-122-5p has been associated with proliferation and metabolic maintenance processes. Interestingly, our KEGG analysis revealed that metabolic, calcium, and RAS signaling pathways are differentially regulated in EVs of IPF cell lines (Figure 6C); this evidence suggests that miR-122-5p regulates several signaling pathways that might influence the lung microenvironment during IPF development, such as the MERK–ERK signaling pathway. Using TGF-α-stimulated cells and a mouse model, the authors also observed that MEK inhibition prevents progression of established fibrosis and provides an attractive target for targeting the MEK pathway in fibrotic lung disease [45], as well as suggests that metabolic pathways are molecular mechanisms closely associated with IPF pathogenesis.

Our analysis also showed that miR-615-3p, miR-181a-2-3p, and miR-143-3p had the lowest expression levels in the IPF cell lines. MiR-615-3p has been found upregulated in disseminated bone marrow tumor cells in patients with hepatocellular carcinoma [46]. Besides, miR-615-3p has also been upregulated in breast cancer cells and tissues, regulating increased cell motility and metastasis [47]. On the contrary, our results show that miR-615-3p is downregulated in IPF, and the KEGG analysis showed that genes regulated by miR-615-3p are associated with cell motility and RNA transport processes, which might be involved in fibrogenesis during IPF development (Figure 6D).

MiR-181a-2-3p has been found downregulated in lung epithelial cells after cadmium exposure, increasing COPD-related inflammatory response, stimulating inflammatory responses as well as bronchial epithelial cell activation [48]. Our results show that this miRNA is also downregulated; since our KEGG analysis showed that it is involved in regulating another biological regulation, such as Hippo signaling pathways, it would be interesting to determine its involvement in IPF (Figure 6E). Interestingly, this signaling pathway has been investigated in respiratory epithelial cells in the lung of IPF patients; for example, it was found that the YAP expression (S127A) in human bronchial epithelial cells increased p-S6 and p-PI3K and drove cell proliferation and migration. In contrast, these processes were inhibited by YAP-TEAD inhibitor; these YAP and mTOR/p-S6 signaling pathways interact to induce cell proliferation and migration and inhibit epithelial cell differentiation that may contribute to IPF pathogenesis [49]. 

The ectopic expression of miR-143-3p has shown tumor suppressor activity in the lung adenocarcinoma cell lines A549 and H1299 [50]. Also, this miRNA has been found downregulated in pulmonary artery smooth muscle cell-derived exosomes (PASMCs), which modulates both migration and angiogenesis [51]. Additionally, miR-143-3p has been found upregulated in patients with acute ischemic stroke (IS), where an RNA-seq analysis revealed three upregulated microRNAs, namely, miR-125a-5p, miR-125b-5p, and miR-143-3p [52]. Moreover, these miRNAs have been proposed as early diagnostics markers. We found that miR-143-3p was downregulated. Our analysis revealed that these miRNAs are predicted to regulate the MAPK signaling pathway, which strongly suggests the involvement of this pathway during fibrogenesis of IPF (Figure 6F).

We also identified other differentially expressed miRNAs, such as miR-138-5p, miR-129-5p, miR-375, and let-7c-5p, suggesting novel research approaches for integrating additional molecular mechanisms regulated by them. For instance, miR-138-3p upregulation inhibits Survivin translation, and it has been proposed as a potential tumor suppressor in bladder cancer. An inverse correlation has also been reported between miR-138-5p expression and Survivin protein levels in bladder cancer tissue samples [53]. miR-129-5p has been found downregulated in NSCLC; however, its upregulation inhibits tumor growth and generates chemoresistance by downregulating DLK1 (delta-like non-canonical Notch ligand 1), which suggest that miR-129-5p and DLK1 may play a relevant role in NSCLC and its treatment [54]. MiR-375 has been proposed to predic early-stage breast cancer, especially in estrogen receptor α (ER-α)-positive patients [55]. Let-7c-5p is downregulated in breast cancer tissues, and its upregulation in MCF-7 breast cancer cell line significantly inhibits cell proliferation and induces cell apoptosis [56]. Although it has been reported that these miRNAs are involved in the progression of different diseases, they have not been analyzed in IPF development. Thus, as other miRNAs identified in this investigation, these miRNAs could be investigated as potential therapeutic targets in the development of IPF. 

## 5. Conclusions

Our results show that miRNAs in EVs isolated from IPF cell lines play a critical role in regulating multiple processes of cell physiology. By analyzing EVs cargo from normal and IPF human lung fibroblasts, we identified miRNA expression patterns that might be useful as either potential disease biomarkers or therapeutic targets. However, further analysis is still needed to elucidate the critical role of the identified miRNAs during the IPF progression.

## Figures and Tables

**Table 1:** Differentially expressed miRNAs of EVs isolated from IPF fibroblasts.

	IPF-1	IPF-2		IPF-1	IPF-2
Upregulated	log2FoldChange	log2FoldChange	Downregulated	log2FoldChange	log2FoldChange
miR-4326	6.9935	6.8393	miR-615-3p	−2.9597	−4.1917
miR-200c-3p	2.9927	2.1989	miR-181a-2-3p	−1.6137	−1.2446
miR-122-5p	2.5596	1.5467	miR-143-3p	−1.0911	−0.99751
miR-122b-3p	2.5596	1.5467	miR-424-3p	−1.0633	−0.98751
miR-129-5p	2.1549	1.8152	let-7c-5p	−0.96183	−1.2443
miR-1299	1.9723	1.7458			
miR-133a-3p	1.6403	1.5317			
miR-148a-3p	1.5762	1.6696			
miR-339-5p	0.98874	1.0852			
miR-1303	0.93803	1.2137			
miR-222-3p	0.79026	1.3043			
miR-221-3p	0.77688	1.0204			

## Data Availability

All data generated or analysed during this study are included in this published article (and its supplementary information files). However, if you need the raw data, please ask to corresponding author.

## Supplementary Materials

Supporting information can be downloaded at: https://www.mdpi.com/article/10.3390/cells11071112/s1, Table S1. Dynamic light scattering measurements (DLS) of EVs from fibroblast. Table S2. Total reads and control quality from samples. Table S3. Novel miRNA. Table S4. miRNA in VEs. Table S5. Readcount TPM. Table S6. Differential analysis IPF1 vs. NL. Table S7. Differential analysis IPF2 vs. NL. Table S8. miRNAs upregulated and downregulated. Table S9. GO enrichment for upregulated miRNA in the pairwise comparison IPF vs. NL by using Genecodis. Table S10. GO enrichment for downregulated miRNA in the pairwise comparison IPF-2 vs. NL by using Genecodis. Table S11. KEGG Pathway for upregulated miRNA in the pairwise IPF vs. NL, using Genecodis. Table S12. KEGG Pathway for downregulated miRNA in the pairwise IPF vs. NL, using Genecodis. Table S13. Target genes of the 3 principal miRNAs upregulated (in common) from IPF. Using the miRWalk and Cytoscape web resource. Table S14. Target genes of the 3 principal miRNAs downregulated (in common) from IPF. Using the miRWalk and Cytoscape web resource. Table S15. KEGG Pathway for DEG-miRNA in the pairwise IPF vs. NL. Using Genecodis.
